# The Columbus Meetings

**Published:** 1899-07

**Authors:** 


*EDITORS:*

THOMAS LOTHROP, M. D. - WM. WARREN POTTER, M. D.

All communications, whether of a literary or business nature, should be addressed to the managing editor: 284 FRANKLIN STREET, BUFFALO, N. Y.

VOL. XXXVIII. JULY, 1899. NO. 12.

### THE COLUMBUS MEETINGS.

THE month of June always witnesses the culmination of the spring medical meetings whenever the American Medical Association pitches its annual meeting north of Mason and Dixon's line. This year Columbus was fixed upon as the place, and the first week in June as the time.

### AMERICAN ACADEMY OF MEDICINE.

As a matter of course a number of other medical organisations always hold meetings in conjunction with the parent society, the most prominent of which is the American Academy of Medicine. The membership in this body is made up of those physicians who also hold academic degrees or their equivalents and its special province is to discuss questions pertaining to medical sociologics. A splendid program was made up by the secretary, Dr. Charles McIntire, of Easton, and the meeting has been designated as one of the most successful in the history of the Academy.

Officers were elected as follows: President, G. Hudson Wakuen, of Philadelphia; vice-presidents, C. G. Plummer, of Salt Lake City; A. Goldspohn, of Chicago; Edwin F. Wilson, of Columbus, and A. L. Benedict, of Buffalo; secretary and treasurer, Charles McIntire, of Easton, Pa.; assistant secretary, W. L. Pyle, of Philadelphia.

### AMERICAN MEDICAL ASSOCIATION.

The fiftieth annual meeting of the American Medical Association convened at the Columbus Opera House, Tuesday, June 6, 1899, under the presidency of Professor Joseph McDowell Mathews, M. D., LL. D., of Louisville, Ky. The proceedings were promptly opened at 10 o'clock with prayer by the Rev. Washington Gladding, D. D. Governor Bushnell followed with a model address of welcome and the mayor of Columbus also made a short speech of a like nature, after which the president proceeded to deliver his annual address. This paper was a model of propriety and deserves to be generally read by the medical profession as well as by such of the laity as have sympathy with medical affairs. Dr. Mathews very properly omitted from his message abstract questions of science, leaving this work to the orators whom the association appoints especially for that purpose and proceeded to deal first with questions of administration, and, second, with matters of public health.

The most important administrative change suggested by the president—namely, that the editor should also be chosen as the secretary of the association was adopted almost without dissent, thus placing the association on a better administrative basis than ever before.

The president's suggestions as to consumption and syphilis were so eminently wise and proper as to receive the universal approbation of the house, which broke forth in a storm of applause as he finished each paragraph.

Dr. Mathews deserves to be congratulated by every American physician, not only upon his most appropriate address, but upon his superb administration from beginning to end. Not within our recollection, and this dates to a somewhat early period in the history of the association, have its affairs been managed so prudently and with such masterful skill and good sense as at Columbus.

The principal sections were very largely attended and the programs were quite full and complete; but the intense heat that prevailed prevented the thorough application to many important subjects that their significance deserved. The section dinners were, generally speaking, dull, if not stupid, and had better be abandoned.

Columbus is a most hospitable city and we have nothing but praise for the manner in which it entertained the association. Public and private hospitalities were lavish and the physicians of Columbus, as well as a large number of the laity, including Ohio's progressive and large-hearted governor, are entitled to the highest commendation for the manner in which the convention was cared for. We wish we could speak in equal terms of approval in regard to the hotels. With the exception of the Chittenden they are not up to the standard one might expect in the capital city of one of our greatest commonwealths. The Great Southern utterly failed to meet such expectations as its pretentious appearance would seem to invite.

We think it is a mistake, in view of the ponderous nature of the meetings of this association, for any city with less than a quarter million of population to undertake its entertainment. On some accounts it would seem that the president's suggestion to locate the association permanently at Washington would be an improvement, but we doubt if the proposition will command the support of a majority for some years. Ultimately we have little doubt that it will prevail.

The exhibits at this meeting were unusually good and the building in which they were held, especially constructed from an appropriate architectural design, was admirably adapted for the purpose. Dr. N. R. Coleman, chairman of the committee on exhibits, deserves great credit for his administration of this department, as well as for much other work of an executive character pertaining to the meeting.

The rotunda of the capitol afforded an admirable registration room and all the section halls were conveniently located to the central administrative department.

By and large the Columbus meeting may be pronounced a conspicuous success from almost every viewpoint and it will require great activity and skilful administration in the future to excel it.

### NATIONAL CONFEDERATION OF STATE MEDICAL EXAMINING AND LICENSING BOARDS.

The ninth annual meeting of this body was held in the senate chamber at Columbus, Monday, June 5, 1899. The president, Dr William Warren Potter, of Buffalo, took the chair at 10.30 a. m. and immediately thereafter Governor Asa S. Bushnell delivered an admirable address of welcome on the part of the State of Ohio. He was followed by Dr. Charles A. L. Reed, of Cincinnati, who performed a like office for the medical profession. These addresses were most ably and eloquently responded to by Dr. Joseph M. Mathews of the executive council, president of American Medical Association. In the course of his response Dr. Mathews said that Kentucky after having gotten rid of all other forms of quackery was now turning its attention to the so-called osteopathists, affirming it would not be long before this latest and worst form of medical chicane was cleared from the confines of the blue grass state.

Dr. N. R. Coleman, of the committee on minimum standards of requirement presented his report, which, in the main, favored the establishment of a high school diploma, or its equivalent, as the necessary prerequisite for admission to the study of medicine. The report specified in detail what the high school standard should mean and was the subject of considerable debate. Finally it was adopted and made a recommendation to the medical colleges of the United States.

Papers were read by Dr. T. J. Happel, of Tennessee, and Dr. B. F. Crummer, of Omaha. A number of others were presented by title.

The following officers were elected for the ensuing year : President, Dr. J. M. McCormack, secretary of the Kentucky State Board of Health; first vice-president, Dr. N. R. Coleman, of Columbus, president of the Ohio State Board of Registration and Examination; second vice-president, Dr. B. F. Crumner, of Omaha; secretary-treasurer, Dr. A. Walter Suiter, of Herkimer. It was decided to meet next year at Atlantic City, N. J., on Monday preceding the meeting of the American Medical Association.

### ASSOCIATION OF AMERICAN MEDICAL COLLEGES.

This association held its annual session in the hall of the house of representatives at Columbus, June 5th, with a large number of delegates and attendance.

Dr. D. M. Hodgson, of Chicago, read a paper on The requirements of the medical curriculum, which provoked considerable discussion. Prof. Hughes, of St. Louis, thought too much technical details should not be offered to the student of general medicine.

Prof. Reynolds, of Louisville, thought the instruction of the medical student should be conducted by trained experts in each subject taught, and that the lecturers should all be specialists in their several departments.

Dr. G. C. Savage, of Nashville, by invitation delivered an address on the condition of medical education under the regulations of the southern College Association. He pointed out the steps which had been taken to bring about the present announcements of the several colleges holding membership in the Southern Association to adopt the standards of the national body, and predicted that all the southern colleges would be in the National Association before 1903. The president delivered a brief, practical address.

About twenty colleges were admitted to membership and the application of some four or more were rejected. Among those admitted was the Medical Department of the University of Buffalo. The association decided that no college can confer the degree of doctor of medicine *ad eundem*, which action is to be commended. The abuses likely to grow up under this privilege are many.

